# Regulating Mid-infrared to Visible Fluorescence in Monodispersed Er^3+^-doped La_2_O_2_S (La_2_O_2_SO_4_) Nanocrystals by Phase Modulation

**DOI:** 10.1038/srep37141

**Published:** 2016-11-15

**Authors:** Qiwen Pan, Dandan Yang, Shiliang Kang, Jianrong Qiu, Guoping Dong

**Affiliations:** 1 State Key Laboratory of Luminescent Materials and Devices and Institute of Optical Communication Materials, School of Materials Science and Engineering, South China University of Technology, Guangzhou, 510640, China; 2 State Key Laboratory of Modern Optical Instrumentation, College of Optical Science and Engineering, Zhejiang University, Hangzhou 310027, China

## Abstract

Rare earth doped mid-infrared (MIR) fluorescent sources have been widely investigated due to their various potential applications in the fields of communication, chemical detecting, medical surgery and so forth. However, with emission wavelength extended to MIR, multiphonon relaxation process that strongly quenched the MIR emission is one of the greatest challenges for such practical applications. In our design, we have described a controllable gas-aided annealing strategy to modulate the phase, crystal size, morphology and fluorescent performance of a material simultaneously. Uniform and monodispersed Er^3+^-doped La_2_O_2_S and La_2_O_2_SO_4_ nanocrystals with a similar lattice structure, crystallinity, diameter and morphology have been introduced to investigate the impact of multiphonon relaxation on luminescence performance. Detailed spectroscopic evolutions in the region of MIR, near-infrared (NIR), visible upconversion (UC) and their corresponding decay times provide insight investigation into the fluorescent mechanism caused by multiphonon relaxation. A possible energy transfer model has also been established. Our results present direct observation and mechanistic investigation of fluorescent evolution in multiphonon relaxation process, which is conductive to design MIR fluorescent materials in the future. To the best of our knowledge, it is the first investigation on MIR fluorescent performance of La_2_O_2_S nanocrystals, which may find various applications in many photoelectronic fields.

Mid-infrared (MIR), usually defined as the spectral range 2~5 μm, covers the most significant high transparency atmospheric window and numerous “molecular fingerprints” of gases, liquids and solids^1^,^2^,^3^. Therefore, MIR sources are of great important for fundamental and practical applications such as optical communications, pollutants detection, trace chemical analysis as well as the next generation imaging devices for remote sensing and medical contrast agents^4^,^5^,^6^,^7^. To date, various radiative emissions in MIR region have been observed in doped systems with rare earth Tm^3+^, Ho^3+^, Er^3+^, Dy^3+^, Pr^3+^ and Tb^3+^ ions^8^,^9^,^10^,^11^,^12^,^13^. Compared to other lanthanide ions, Er^3+^ ion is one of the most efficient MIR activators excited by commercial near-infrared (NIR) 980 nm laser via ground state absorption of ^4^I_15/2_ → ^4^I_11/2_. Its 2.6~2.7 μm emission from transition of ^4^I_11/2_ → ^4^I_13/2_ is closed to the O-H vibration peak at about 3 μm and therefore can be strongly absorbed by biological tissues, which is attractive in medical surgery and eye-safe laser radar^14^,^15^,^16^.

Extending rare earth transitions into MIR, the excited state electrons tend to relax to lower levels nonradiatively by multiphonon relaxation due to the decreasing energy gap. Therefore, MIR transition rate is governed by the maximum phonon energy of host lattice. It is known that crystallinity, doping concentration, dopants’ substitution sites in the host strongly modified their MIR performance^17^,^18^,^19^,^20^. In order to investigate the impact of multiphonon relaxation on luminescence performance, we present a direct comparison and mechanistic investigation on the luminescence evolution of La_2_O_2_S (LOS) and La_2_O_2_SO_4_ (LOSO), which have similar lattice structure, crystallinity, size and morphology.

Rare earth doped lanthanide oxysulfide RE_2_O_2_S and oxysulfate RE_2_O_2_SO_4_ are outstanding luminescent phosphors for applications. RE_2_O_2_S with stable thermal and chemical properties, high refractive index (~2.2)^21^, wide band-gap and low average phonon energy (~520 cm^−1^)^22^ has become an efficient phosphor for visible and NIR emission^23^,^24^,^25^. For UC emission, RE_2_O_2_S with optimized Yb^3+^/Er^3+^ doping ratio has shown a comparable or even higher quantum yield with respect to the well-known brightest NaYF_4_ at a reasonable low excitation density^26^,^27^,^28^,^29^. Their NIR emission is also demonstrated with measured quantum yield higher than 100% in Er^3+^/Yb^3+^ co-doped La_2_O_2_S^30^. Lanthanide oxysulfate RE_2_O_2_SO_4_ also displays a unique fluorescent property activated by rare earth (Eu^3+^, Er^3+^, Ce^3+^ and so forth)^31^,^32^,^33^. Eu^3+^: Y_2_O_2_SO_4_ offers 3 times stronger emission compared to the its oxide counterpart Eu^3+^: Y_2_O_3_^34^.

It is worth noticing that RE_2_O_2_S and RE_2_O_2_SO_4_ adopt a similar crystal structure^35^,^36^. The phase transition between RE_2_O_2_S and RE_2_O_2_SO_4_ in fact is the removal or insertion of oxide anions surround sulfur^37^. In our design, by modulate the phase transition simply through a gas-aided annealing, we synthesized monodispersed 5% Er^3+^-doped LOS and LOSO nanocrystals by the same hydrothermal precursor with similar crystallinity, size and morphology in order to investigate the impact of multiphonon relaxation on luminescence performance. The effect of gas-aided phase modulation on the morphology and fluorescence performance of the resulted LOS/LOSO nanocrystals was also discussed. We present a detailed spectroscopic investigation on Er^3+^-doped LOS and LOSO in their MIR, NIR and visible UC emission and corresponding decay times. An intuitive relationship between multiphonon relaxation with fluorescent evolution in the emission region ranging from MIR, NIR to visible UC was established. To the best of our knowledge, it is the first investigation on MIR emission of LOS and LOSO, which have great potential to develop various novel applications in many photoelectronic fields.

## Results

### Phase and morphology characterization

**Figure 1:**
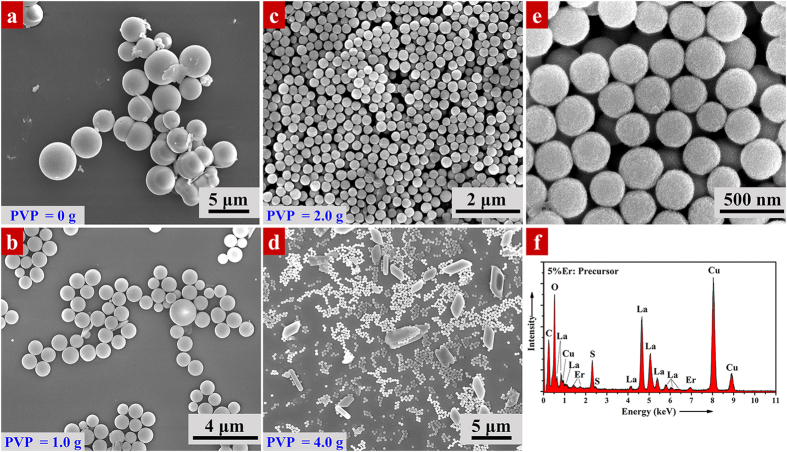
SEM images of 5% Er^3+^-doped hydrothermal precursors synthesized with different amount of PVP: (**a**) 0 g PVP; (**b**) 1.0 g PVP; (**c**) 2.0 g PVP and (**d**) 4.0 g PVP. (**e**) High magnification SEM images of as-prepared 5% Er^3+^-doped precursors obtained with 2.0 g of PVP. (**f**) EDS spectrum of the corresponding 5% Er^3+^-doped precursor prepared with 2.0 g of PVP. The signal of Cu comes from the copper grid.

It is reported that PVP plays an important role in control the morphology and size of nanoparticles. To shed more light on the effect of PVP, Fig. 1 displays the SEM images of 5% Er^3+^-doped hydrothermal precursors synthesized with different amount of PVP: (a) 0 g PVP; (b) 1.0 g PVP; (c) 2.0 g PVP and (d) 4.0 g PVP. Most of the precursors are spherical, driven by the minimization of interfacial energy in hydrothermal process. As depicted in Fig. 1(a), precursors without PVP are spherical, but agglomerate with a large diameter of 2~3 μm. However, when PVP is introduced, the resulted particles become highly monodispersed with an average diameter of 900~1000 nm for PVP = 1.0 g (Fig. 1(b)) and ~300 nm for PVP = 2.0 g (Fig. 1(c)). Increasing the amount of PVP to 4.0 g, the resulted sample shows two distinct sizes and morphologies: a small spherical particle in 300 nm and a large nanosheet in 2~3 μm (Fig. 1(d)). The following three unique intrinsic natures of PVP determine the shape evolution of resulted samples: (1) PVP molecule contains both hydrophilic and hydrophobic groups. It is reported that PVP can strongly coordinated with rare earth ions by its O and N atoms in the pyrrolidone rings^23^. (2) The hydrophobic carbon chains in PVP can prevent nanocrystals agglomerate via repulsion attributed to the steric hindrance effect extended to the solvent^38^. Therefore, once PVP introduced into the reaction, PVP will cover on the surface of molecules to slow down the growth rate and prevent self-aggregation of nanocrystals. When PVP increased from 1.0 g double to 2.0 g, the steric hindrance is more effective, resulting in a diameter reduction from 900~1000 nm to ~300 nm. (3) PVP is a morphology controller which will modify the crystal growth in a specific lattice facet^39^. With increasing PVP up to a critical level, the surface free energy of a specific facet becomes different to others. Spherical morphology can no longer to sustained, leading to the formation of nanosheet. Figure 1(e) shows the high magnification SEM image of as-prepared 5% Er^3+^-doped precursors obtained with 2.0 g of PVP. With a closer examination, the spherical particles are highly uniform with a smooth surface. EDS spectrum (Fig. 1(f)) reveals that the compounds contain elements of La, O, S and Er. The peak of C is a part from the organic molecular in precursor, and a part from the copper grid. The signal of Cu only comes from the copper grid. No other impurities are found in the EDS spectrum. Therefore, we concluded that PVP = 2.0 g is the optimal amount of surfactant for this hydrothermal reaction.

**Figure 2:**
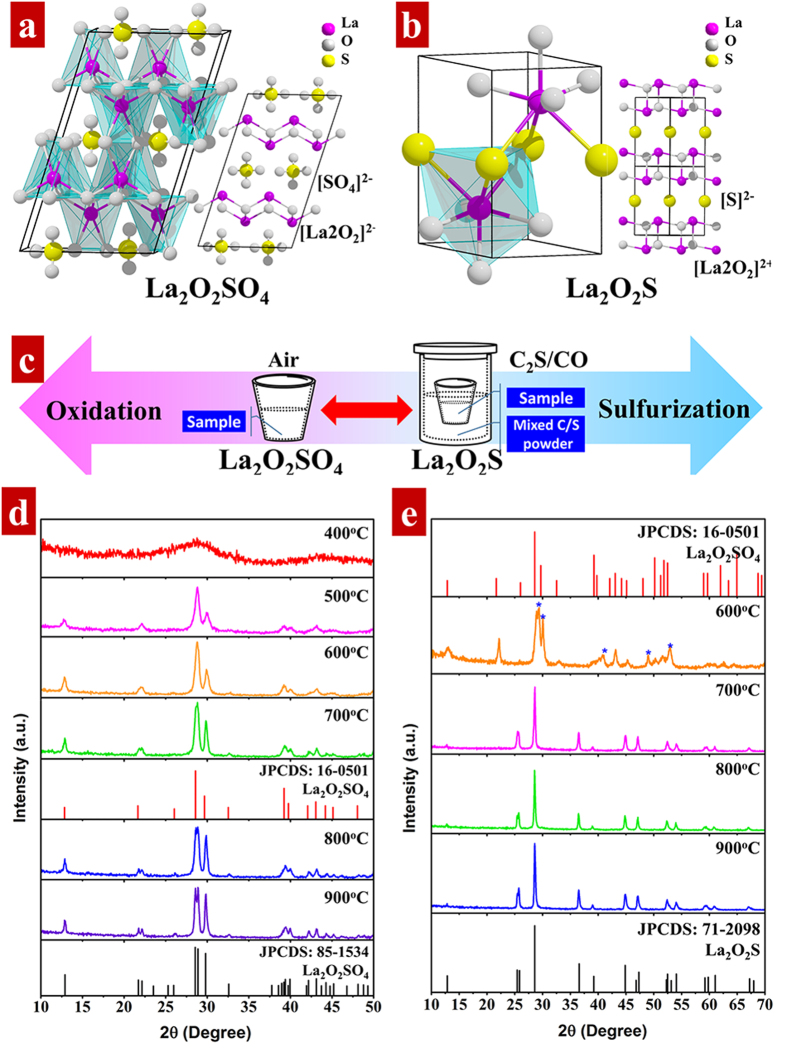
Schematic presentations of (**a**) La_2_O_2_SO_4_ and (**b**) La_2_O_2_S crystal structure. (**c**) Illustration of process of heat treatment of hydrothermal precursor to synthesis La_2_O_2_S and La_2_O_2_SO_4_. XRD patterns of (**d**) La_2_O_2_SO_4_ and (**e**) La_2_O_2_S calcined at different temperatures for 2 h in the atmosphere of air and CS_2_/CO, respectively.

LOSO and LOS adopt a similar crystal structure^35^,^36^. Both LOSO and LOS crystal structures are consist of two alternative stacking units: a positive charged (La_2_O_2_)^2+^ layer and a layer of anion groups, sulfate (SO_4_)^2−^ for LOSO (Fig. 2(a)) and sulfide (S^2−^) for LOS (Fig. 2(b)). The stacking layers provide large lattice constants which is beneficial for subsequent doping. In a layer of (La_2_O_2_)^2+^, each La atom coordinated with four oxygen anions, which can be considered as a basic unit (LaO_4_). Then the (LaO_4_) units connect each other to form a two-dimensional (La_2_O_2_)^2+^ framework by sharing edges. For LOSO, in addition to four oxygen atoms in (La_2_O_2_)^2+^ layer, La atoms are also coordinated with the other two oxygen anions belonging to two different (SO_4_)^2−^, leading to a six-coordinated geometry in La-O bonding. For LOS, the layers of (SO_4_)^2−^ in LOSO are replaced by layers of S^2−^. La atoms are bonded with three S^2−^ anions to produce a seven-coordinated geometry with four oxygen anions in the (La_2_O_2_)^2+^ plane. The coordination determines the optical spectrum of the compound, which will be discussed in detail later. Such a structural similarity reveals that phase transition between LOSO and LOS is the insertion or removal of oxide anions surround sulfur^37^. Therefore, we reasoned that calcination of sulphuric precursors in oxide/sulfurized atmosphere can produce pure LOSO and LOS. And we choose a facile controllable gas-aided sulfur treatment to prepare LOS and LOSO, as depicted in Fig. 2(c).

Figure 2(d,e) show the XRD patterns of LOSO and LOS calcined at different temperatures for 2 h in the atmosphere of air and CS_2_/CO, respectively. For LOSO, XRD pattern of hydrothermal precursor calcined at 400 °C shows a broad featureless peak, indicating the amorphous or non-crystalline nature of the initial powder. Elevating the annealing temperature to 500~600 °C, the XRD patterns can be indexed to the standard tetragonal structural of LOSO (JPCDS: 16-0501) and no other impurities were observed. Phase transition from tetragonal LOSO to monoclinic is evident in 700 °C, as the diffraction signal located at ~22° split into two adjacent peaks. Further increased the annealing temperature above 800 °C, all the diffraction peaks of LOSO can be indexed to the standard monoclinic LOSO phase (JPCDS: 85-1534), indicating a complete transformation from tetragonal to monoclinic phase. For LOS, the XRD pattern of hydrothermal precursors calcined at 600 °C can be indexed to the tetragonal LOSO. The peaks broadening is due to the poor crystallinity at 600 °C. After the temperature increased to 700~900 °C, the precursors are all converted to the standard hexagonal phase of LOS (JPCDS: 71-2098), and no extra impurities were indexed. Therefore, it is confirmed that LOS is the result of sulfidation of LOSO. Both the diffraction peaks of LOSO and LOS become sharp and intense with annealing temperature increased, indicating a better crystallinity after annealing.

The thermal decomposition behavior of the hydrothermal precursor was investigated by thermogravimetric analysis (TG-DSC) conducted from room temperature to 1000 °C with a heating rate of 10 °C/min in air. The TG curves (Supplementary Fig. S1) shows a three-step weight loss ranging from 25 °C~700 °C with a total weight loss of ~22.75%. The first step ranging from 25 °C to 250 °C with a mass loss of ~4.41% is attributed to the removal of water and organic decomposition. With temperature increasing to ~520 °C, the second obvious weight loss of ~15.39% is found, corresponding to two exothermic peaks at ~327.3 °C and ~529.8 °C in the DC curve. Pure PVP is oxidized at ~420 °C in air^40^. However, the decomposition temperature will decrease when PVP bonding with other metals in different experiment condition. Li *et al*. reported the oxidation of PVP bonded with Ag starts from 250 °C and ended at ~400 °C, which is similar to our TG data^41^. Therefore, we reasoned that the first exothermic peak at ~327.3 °C is due to the decomposition of PVP in hydrothermal precursors. The second exothermic peak is related to crystallization process of LOSO at ~500 °C, which is consistent with our XRD patterns in Fig. 2(d). The third step at higher temperature in TG curve corresponds to an exothermic peak at ~732.3 °C in DSC, which is related to the phase transformation from tetragonal to monoclinic according to our XRD results. The slight weight loss is due to the decomposition of residue organic ligand. A flat platform is formed at above 730 °C indicating that a stable compound has been achieved. Because the gas-aided sulfurization process is not able to perform in the simultaneous thermal analyzer, thereby we selected 700~900 °C for both the annealing temperature of LOS and LOSO.

**Figure 3:**
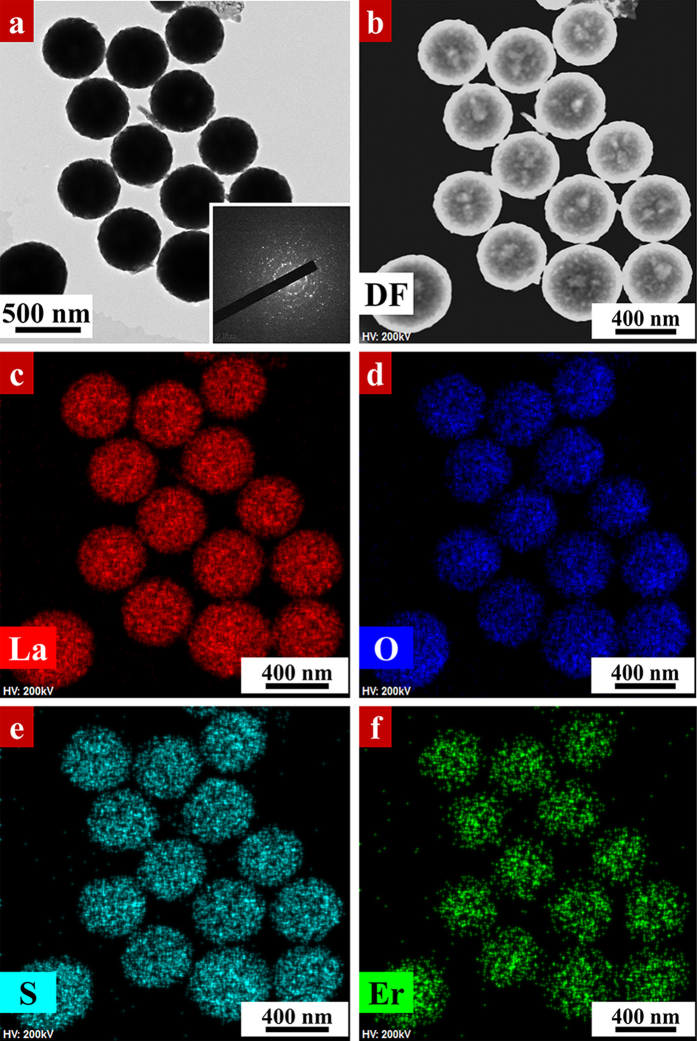
(**a**) TEM image of 5% Er^3+^-doped La_2_O_2_SO_4_ nanocrystals calcined at 700 °C. (**b**–**f**) HAADF STEM and elemental maps of (**c**) La, (**d**) O, (**e**) S and (**f**) Er in 5% Er^3+^-doped La_2_O_2_SO_4_ nanocrystals.

**Figure 4:**
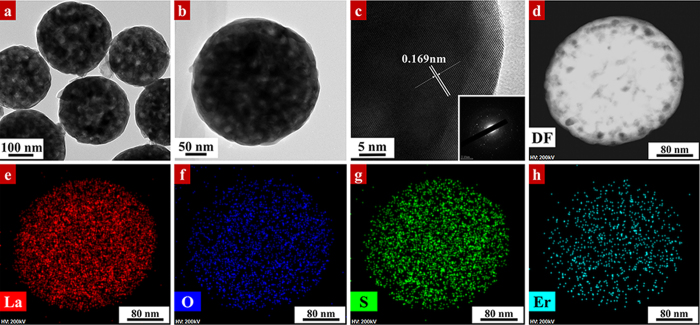
(**a**) Low-magnification, (**b**) high-magnification TEM and (**c**) HRTEM image images of 5% Er^3+^-doped La_2_O_2_S nanocrystals calcined at 700 °C. (**d**–**h**) HAADF STEM and elemental maps of (**e**) La, (**f**) O, (**g**) S and (**h**) Er in 5% Er^3+^-doped La_2_O_2_S nanocrystals.

Figure 3 shows the TEM characterization of LOSO nanocrystals calcined at 700 °C in air. As depicted in Fig. 3(a), the resulted LOSO is completely solid spherical with an average diameter of ~300 nm. Elemental maps of the 5% Er^3+^-doped LOSO in Fig. 3(c–f) reveals that elements of La, O, S and Er distributed homogeneously in LOSO particles, which is consistent with our EDS spectrum (Supplementary Fig. S2). The morphology and size of LOS nanocrystals in Fig. 4(a,b) are similar to that of in LOSO, as depicted in Fig. 3. The HRTEM (Fig. 4(c)) images show the distance between two adjacent lattice fringe is 0.169 nm, corresponding to the (021) crystal plane of LOS. The elemental maps of 5% Er^3+^-doped LOS in Fig. 4(e–h) demonstrated a homogeneous distribution of La, O, S and Er elements. This confirms that Er^3+^ ions are successfully doped into LOS particles through our synthetic strategy. Detailed EDS spectrum also illustrated the existence of the above elements in LOS nanocrystals (Supplementary Fig. S3).

**Figure 5:**
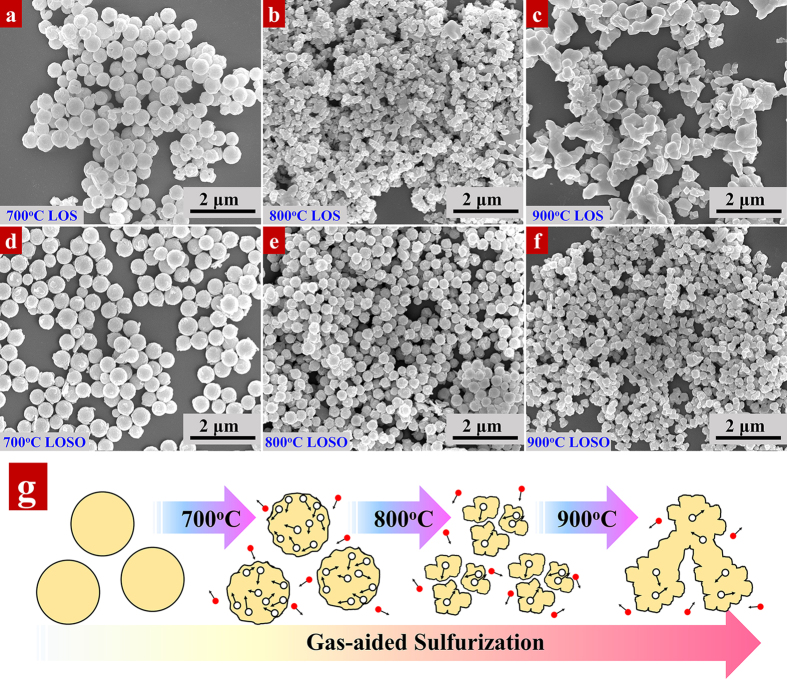
(**a**–**c**) SEM images of 5% Er^3+^-doped La_2_O_2_S nanocrystals calcined under CS_2_/CO atmosphere at (**a**) 700 °C, (**b**) 800 °C and (**c**) 900 °C, respectively. (**d**–**f**) SEM images of 5% Er^3+^-doped La_2_O_2_SO_4_ nanocrystals annealed under air flow at (**d**) 700 °C, (**e**) 800 °C and (**f**) 900 °C, respectively. (**g**) Schematic of morphology evolution with increasing annealing temperature in Er^3+^-doped La_2_O_2_S.

Figure 5 shows the SEM images of LOS and LOSO nanocrystals converted from the same hydrothermal precursor with a controllable gas-aided process at temperature ranging from 700 °C to 900 °C. It is obvious that both LOS (Fig. 5(a)) and LOSO (Fig. 5(d)) maintain good spherical shape of precursors with an average diameter of ~300 nm after annealing at 700 °C, which is consistent with the TEM images. Their surface becomes relatively rougher due to removal of substantial PVP, as mentioned before in the thermal analysis. However, further increasing the annealing temperature to 800 °C and 900 °C, the morphology changed differently in LOS and LOSO. For LOS, nanocrystals split into smaller nanoparticles at 800 °C (Fig. 5(b)), then agglomerate and grow up at 900 °C (Fig. 5(c)). For LOSO, the particles maintain good spherical morphology with a slight diameter decrease at 800 °C (Fig. 5(e)) due to the removal of slight organic residues, and then start to agglomerate into a network at higher temperature of 900 °C (Fig. 5(f)). The excellent thermal stability shows that both LOS and LOSO have great potential to composite with glass^42^. The morphology evolution has great influence on the fluorescent performance of the nanocrystals, which will be discussed later in detail.

The effect of annealing on crystal morphology is schematically depicted in Fig. 5(g). In general, the formation mechanism of the nanocrystals can be artificially divided into two growing process, in which assembled amorphous precursors are nucleated first, and then nanoparticles grow by Ostwald ripening and form larger nanocrystals. The oxidation process of LOSO in air is a complete reaction from inner to outward, thereby they could maintain good spherical shape of the as-prepared precursors. However, for LOS, the situation is rather different. Two interaction processes are included during sulfidation: the phase transformation from LOSO to LOS firstly, and then the disruption from large nanocrystals to fine particles. In the initial stage, the transformation from LOSO to LOS is related to a nanoscale Kirkendall process, which refers to a mutual non-equilibrium diffusion process through vacancies exchange in the interface of a material^43^,^44^,^45^,^46^. The Kirkendall effect suggests the generation of nanoscale voids, as a type of defects, promoting the disruption of large nanocrystals at 800 °C. Under the atmosphere of CS_2_/CO, sulfur atoms tend to diffuse into the particles, react with surface LOSO through the gas-solid interface and carry the oxygen atoms out of the crystals simultaneously. It is expected that the diffuse rate of LOS in LOSO solid is faster than that of atmospheric sulfur during sulfidation, leading to the formation of Kirkendall voids. In other words, the LOS nanocrystals are assembled by primary smaller nanocrystals. In the second stage, when the sintering temperature is higher than 800 °C, defects in materials will dramatically increase driven by thermal dynamics, LOS nanocrystals tend to split up into the primary smaller particles. With the annealing temperature increased, small LOS nanocrystals tend to agglomerate and recrystallization in order to minimize the interfacial energy at higher temperature.

### MIR, NIR and visible UC fluorescence in LOS and LOSO

**Figure 6:**
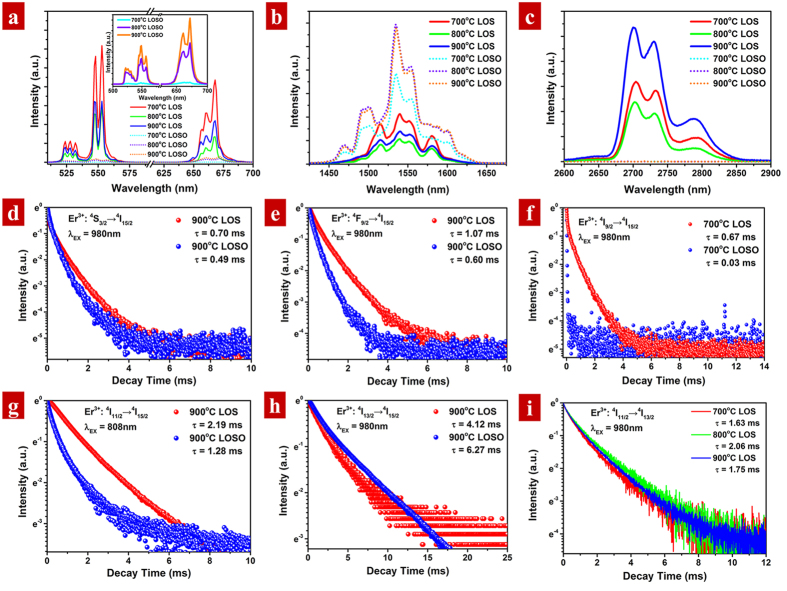
(**a**–**c**) The emission spectra of (**a**) visible upconversion, (**b**) near infrared and (**c**) mid-infrared emission in 5% Er^3+^-doped La_2_O_2_S and La_2_O_2_SO_4_ nanocrystals calcined at 700 °C–900 °C with an excitation of 980 nm LD, respectively; (**d**–**h**) Fluorescence decay curves of (**d**) Er^3+^: ^4^S_3/2_ → ^4^I_15/2_, (**e**) Er^3+^: ^4^F_9/2_ → ^4^I_15/2_, (**f**) Er^3+^: ^4^I_9/2_ → ^4^I_15/2_, (**g**) Er^3+^: ^4^I_11/2_ → ^4^I_15/2_ and (**h**) Er^3+^: ^4^I_13/2_ → ^4^I_15/2_ upon pulse LDs of 980 nm and 808 nm, respectively; (**i**) Mid-infrared (Er^3+^: ^4^I_11/2_ → ^4^I_13/2_) decay curves of 5% Er^3+^-doped La_2_O_2_S calcined at 700–900 °C upon pulse LD of 980 nm, respectively.

Efficient fluorescent emissions in visible UC, NIR and MIR emission of 5% Er^3+^-doped LOS and LOSO calcined at 700 °C~900 °C upon continuous excitation of 980 nm LD are displayed in Fig. 6. In the visible UC emission spectra in Fig. 6(a), three emission bands located at ~530 nm, ~550 nm and ~660 nm are observed in both LOS and LOSO, which is assigned to Er^3+^ transition from the ^2^H_11/2_, ^4^S_3/2_ and ^4^F_9/2_ excited state to the ^4^I_15/2_ ground state, respectively. In the NIR emission spectra (Fig. 6(b)), five overlapped peaks locate at 1469, 1497, 1535, 1544 and 1588 nm are composed of the ^4^I_13/2_ → ^4^I_15/2_ emission band in LOSO. However, only three major overlapped peaks centered at 1515 nm, 1540 nm, 1552 nm and 1581 nm are found in the spectra of LOS. For MIR emission spectra (Fig. 6(c)), it is worth noticing that such emission is only exhibited in the host of LOS. The surprised absence of MIR emission in LOSO will be discussed later in detail. Three peaks centered at ~2700 nm, ~2730 nm and ~2790 nm are observed in the MIR spectra of LOS, which is assigned to the Er^3+^: ^4^I_11/2_ → ^4^I_13/2_ transition. It is obvious that the spectral shape and peak position is similar in a host with different annealing temperatures, indicating that the pure phase of both LOS and LOSO calcined at 700~900 °C, which is consistent with our XRD results. However, the Stark splitting of Er^3+^ optical active centers in LOS and LOSO is rather different, which may due to the different polyhedral symmetry of Er^3+^ ions in LOS and LOSO.

It is reported that morphology plays a significant role in fluorescence properties in nanoscale. To better understand the relationship between morphology, annealing schedule and fluorescence, we compared the spectral intensity (Fig. 6) of visible UC, NIR and MIR emission at different temperatures of 700 °C~900 °C in the same host. For all the samples of LOSO, the emission intensity increased with the annealing temperature elevated, that is 900 °C > 800 °C > 700 °C. The slight difference in NIR spectra can be attributed to the instrumental error. In contrast, increased the annealing temperature of LOS from 700 °C to 900 °C, the fluorescent performances for all optical region show an abnormal diminish at 800 °C, and then recover at 900 °C. It is known that annealing can increase the fluorescent luminescence due to the following three reasons^47^: (1) removing the organic residues which serves as recombination centers that normally induce quenching in fluorescence; (2) diminishing defects or other lattice distortion in crystals through thermal diffusion; (3) agglomerate small crystals with size growth. The last reason affects the morphology of nanocrystals, which is the most important for fluorescence enhancement.

For LOSO, according to the SEM images depicted in Fig. 5(d–f), the diameter of the resulted samples decreased slightly with increasing temperature. The tiny reduce of diameter is attributed to the removal of organic residues in the crystal, according to the thermal analysis. Therefore, fluorescent performance will be enhanced with increasing annealing temperature as the reasons listed above. In addition, the monoclinic phase of LOSO may have a better luminescence performance than that of the hexagonal phase, which also leads to an emission enhancement. In contrast, since no phase transformation occurs in LOS during sulfidation at the temperature of 700 °C~900 °C, the morphology evolution must be taken into consideration. According to the SEM analysis, the nanocrystals disrupt into smaller nanoparticles due to the Kirkendall effect at the temperature of 800 °C. The surface to volume ratio dramatically increase, resulting in an abnormal luminescence quenching compared to LOS calcined at 700 °C. However, there is still no general argument on size-dependent luminescence quenching. It may be attribute to the atomic-scale mechanism related to the surface ligand or defect^48^,^49^. Further increasing the annealing temperature to 900 °C, the crystals re-grow with small particle connection. The energy gap of MIR emission is ~3700 cm^−1^, which is much lower than that of visible UC or NIR emission, thereby MIR emission is relatively more sensitive to the defect quenching in crystal. Thus, the MIR emission in 900 °C LOS is stronger than that calcined at 800 °C.

### Effect of multiphonon relaxation on fluorescent emission

Crystal host plays a significant role in the luminescence doping systems. The comparison of optical spectra of visible UC, NIR and MIR between 5% Er^3+^-doped LOS and LOSO were also demonstrated in Fig. 6. As depicted in visible UC emission spectra in Fig. 6(a), the emission intensity in 5% Er^3+^-doped LOS is ultra-strong, which is more than one order of magnitude higher than that in the LOSO samples. However, the situation is inversed in NIR emission (Fig. 6(b)). An increasing intensity by a factor of 3 is measured in the NIR emission spectra for 5% Er^3+^-doped LOSO compared to the LOS samples. In the MIR emission spectra (Fig. 6(c)), emission is only observed in LOS. No emission is found in the LOSO samples. Such fluorescent evolution may be attributed to the multiphonon relaxation, which plays an important role in the fluorescent performance in crystal host.

**Figure 7:**
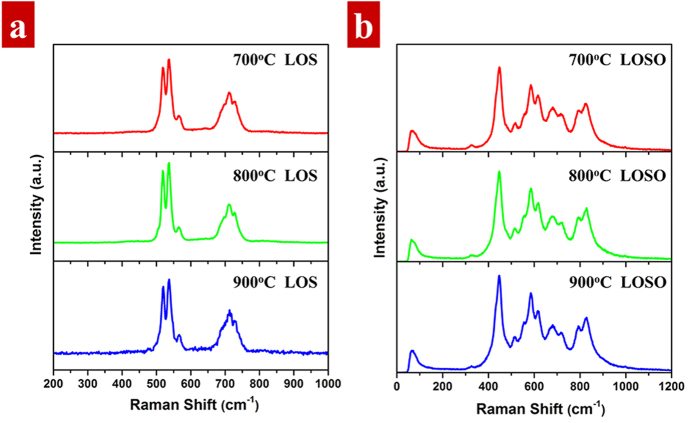
Raman spectra of 5% Er^3+^-doped (**a**) La_2_O_2_S and (**b**) La_2_O_2_SO_4_ calcined at 700–900 °C upon excitation of 532 nm laser.

Raman spectrum is considered as a fingerprint to measure the phonon distribution of materials. To investigate the maximum phonon energy of LOS and LOSO, Fig. 7(a,b) show the Raman spectra of 5% Er^3+^-doped LOS and LOSO calcined at different temperature ranging from 700 °C to 900 °C, respectively. Herein, utilizing a 532 nm excitation source, Er^3+^ dopants could lead to f-f transitions in the region above 1200 cm^−1^. Therefore, the fluorescent signal from Er^3+^ ions will not interfere in our Raman spectra. As depicted in Fig. 7(a), a major band at about 520 cm^−1^ is observed with a shoulder on higher frequency at 568 cm^−1^ in LOS. The principle band is reported associated with sulfur. A smaller band at 710 cm^−1^ is also found in LOS, corresponding to its maximum phonon vibration. The Raman bands are consistent with that reported by Sotnikov *et al*.^50^ For LOSO in Fig. 7(b), a continuous broad band between 450 cm^−1^ to 830 cm^−1^ is composing of the overlapping bands located at 448, 585, 678 and 831 cm^−1^, respectively. The Raman peak at 448 cm^−1^ is assigned to the La-O fundamental mode. The maximum phonon energy is about ~830 cm^−1^ with a long tail up to ~1000 cm^−1^ for LOSO.

The difference in phonon frequency between LOS and LOSO can attribute to the lattice structure. The maximum phonon vibration significantly depends on the coupling between the electronic levels of Er^3+^ and the vibration modes of nanocrystal^51^. As mentioned before, LOS is structural similar to LOSO, with an S layer replaced by the SO_4_^2−^ layer. The covalency of chemical bonds forming LOS is higher than those of SO_4_^2−^ in LOSO. Therefore, it is reasonable that the high vibration of SO_4_^2−^ is contributed to higher maximum phonon energies in the host. In addition, the symmetry of Er^3+^ is also affect the highest phonon vibration mode^52^. The coordination number of Er^3+^ in LOS polyhedral is seven while the number in LOSO is six. Er^3+^ dopants with lower symmetry and higher covalent bond results in high oscillator strength, leading to low phonon energy of LOS. Furthermore, the different crystal splitting in emission spectra are also due to the presence of different sites of Er^3+^ in the host lattice, as mentioned above.

In the multiphonon relaxation process, lattice vibration with maximum phonon energy is considered the most energetic nonradiative quenching, although lower phonon frequency modes cannot be excluded from participation. In a multiphonon relaxation process, the higher the phonon frequency, the larger the probability in multiphonon relaxation quenching is. The probability for the multiphonon relaxation W_MPR, *if*_ between the initial *i* to the final *f* states was found empirically to follow the so called “energy gap law”^19^,^53^:


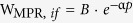


here, B and α are material parameters and bonding to the lattice. P is defined as the number of phonons that need to bridge the energy gap ΔE_q_. The relationship between P and ΔE_q_ is 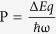, where *ћω* is the corresponding maximum phonon energy of the host. Therefore, multiphonon relaxation will fluctuate exponentially with both the maximum phonon energy in the host and energy gap between the initial to the final state. Multiphonon relaxation becomes dominate if five or less phonons are needed to bridge the gap^53^,^54^. Neighboring energy levels will dominate such relaxation. The most important gap here for Er^3+^ luminescence is ^4^I_11/2_ level, the upper level for MIR emission, which is reported dominate over 97% of the visible UC emission^26^. The gap between ^4^I_11/2_ to the upper ^4^I_9/2_ level is ~2200 cm^−1^, and to the lower level ^4^I_13/2_ is ~3700 cm^−1^, which is directly related to the MIR emission. Therefore, expect for the ^4^I_9/2_ → ^4^I_11/2_, different Er^3+^ transitions could take place within LOS because the energy gap is larger than 5 phonons. When phonon energy larger than 800 cm^−1^, the fluorescence will occur only for energy gaps larger than ~4000 cm^−1^ (<~2400 nm), thus no MIR emission is observed in LOSO, and its total quantum yield is relatively low.

Multiphonon relaxation is a rapid process, shortening the lifetime of an intermediate level. To investigate the effect of multiphonon relaxation on interionic interaction of Er^3+^ ions, we measured the emission decay times of the ^4^S_3/2_, ^4^F_9/2_, ^4^I_9/2_, ^4^I_11/2_ and ^4^I_13/2_ states in LOSO and LOS calcined at the same temperature of 900 °C, as depicted in Fig. 6(d–h). Except for ^4^I_13/2_ → ^4^I_15/2_, lifetimes of LOS are much longer than that of in LOSO. With increasing W_MPR_ in the host, the radiation transitions in system will compete with more and more nonradiative process, leading to a total transition rate decrease. It should be also notice that the ^4^I_9/2_ UC decay curves exhibits an almost immediate decay (0.03 ms) in LOSO compared to that in LOS (0.67 ms, Fig. 6(f)), which is responsible for the emission intensity variation. Upon excitation of 980 nm, the following ground state absorption (GSA) and excited state absorptions (ESA) are possible to occur:


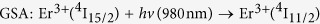



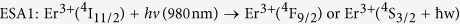



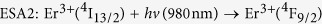


For GSA process in LOSO, the ^4^I_11/2_ population is fed via the ^4^I_9/2_ level by a rapid depletion through nonradiative process. In addition, multiphonon relaxation process also occurs in ^4^I_11/2_ → ^4^I_13/2_ transition, thus most of the photons in GSA are accumulated in the ^4^I_13/2_ levels, yielding relatively strong NIR emission at 1530 nm in LOSO. Lifetime of the intermediate level determines the occupation of higher energy levels. Since the lifetime in ^4^I_11/2_ in LOSO is shorter than that of in LOS, the UC emission will also decrease.

One can found that the intensity ratio of the red to green (G/R) in the UC emission in LOS is G/R > 1, while the situation is inverse in LOSO (G/R < 1). In the ESA1 process, photons excited to ^4^F_9/2_ levels will undergo fast relaxation to the lower ^4^S_3/2_ due to the multiphonon relaxation, populating the red emission in ^4^F_9/2_ level. In addition, since the lifetime of intermediate energy level ^4^I_13/2_ is almost 6 times longer than that in ^4^I_11/2_, photons are also tend to excite to ^4^F_9/2_ levels through ESA2, leading to the enhancement of red emission in LOSO. Besides GSA and ESA, energy transfer upconversion (ETU) between neighboring Er^3+^ ions are also strongly governed the populations of the ^4^S_3/2_ and ^4^F_9/2_. The following ETU processes are observed to occur upon 980 nm excitation:


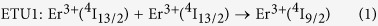



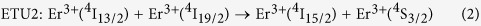



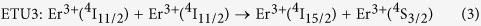


Therefore, increasing the multiphonon relaxation rate can increase the population ratio of the ^4^I_13/2_ to ^4^I_11/2_ levels, thereby enhance the red UC emission and also suppress the green one simultaneously.

## Discussion

**Figure 8:**
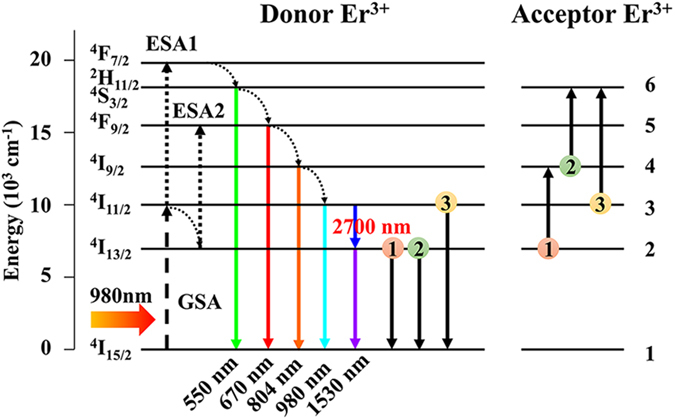
Schematic of possible energy transfer mechanism in Er^3+^-doped La_2_O_2_S/La_2_O_2_SO_4_ with an excitation of 980 nm laser diode. The dashed, dashed-dotted, curved and colored arrows represent ground state absorption, excited state absorption, multiphonon relaxation and fluorescent emission processes, respectively. The proposed energy transfer processes between neighboring Er^3+^ ions are labeled indices from 1 to 3.

The Er^3+^ ions energy levels as well as the possible energy transfer mechanism explaining the Er^3+^ fluorescence in 5% Er^3+^-doped LOS and LOSO are shown in Fig. 8. For LOS, after excitation upon 980 nm, Er^3+^ ions are promoted to the ^4^I_11/2_ levels through ground state excitation. With relatively low phonon frequency, population of Er^3+^ ions to higher excited states upon excitation of 980 nm can be realized by the following two processes: (1) direct ESA of ^4^I_11/2_ → ^4^F_7/2_ or ^4^I_13/2_ → ^4^F_9/2_; (2) ETU processes as mentioned above, in which an Er^3+^ ions can be promoted to ^4^S_3/2_ or ^4^I_9/2_ by consuming the photons in ^4^I_11/2_ or ^4^I_13/2_, respectively. Considered the relatively short lifetime of the ^2^I_11/2_ (1.6~2 ms) compared to the ^4^I_13/2_ level (~4 ms), and the depletion by MIR emission at ~2700 nm, it is clear that ESA2 process ^4^I_13/2_ → ^4^F_9/2_ dominate the ESA process. Therefore, the majority of green UC emission is originated from the ETU process. In general, there is a competition between the various processes. The ETU1 process: ^4^I_13/2_, ^4^I_13/2_ → ^4^I_15/2_, ^4^I_9/2_ followed by the ^4^I_9/2_ → ^4^I_11/2_ multiphonon relaxation is responsible for the main ^4^I_11/2_ population except the LD pumped. Since lifetime of the upper level ^4^I_11/2_ is shorter than that of ^4^I_13/2_ due to the so-called “self-terminating” transition, ETU1 process provide an energy recycle which is benefit for MIR emission: (1) photons with 1530 nm NIR radiation fast deplete to ^4^I_13/2_, which suppress the photons accumulation at lower level of MIR emission; (2) populated ^4^I_9/2_ state will decay to ^4^I_11/2_ through subsequent multiphonon relaxation, leading to an energy recycle for ^4^I_11/2_ → ^4^I_13/2_. Consider the short-lived ^4^I_9/2_ level, the ETU2 process ^4^I_13/2_, ^4^I_9/2_ → ^4^I_15/2_, ^4^S_3/2_ is not very efficient. Since ESA2 dominates the ESA process, thus the ETU3 process ^4^I_11/2_, ^4^I_11/2_ → ^4^I_15/2_, ^4^S_3/2_ followed by multiphonon relaxation process with the assistant of three or four phonons due to the energy mismatch (~2000 cm^−1^) is the main population of the green emission in ^4^F_7/2_ and ^2^H_11/2_/^4^S_3/2_ states. The depopulation of the upper state ^4^I_11/2_ suppresses the MIR fluorescence and should be prohibited in our future work.

For LOSO, with continuous phonon frequency more than 800 cm^−1^, the total quantum yield is relatively low and the fluorescent mechanism is rather different. After excitation of 980 nm, the Er^3+^ ions are promoted to the ^4^I_11/2_ state. With a dynamic multiphonon relaxation, the ESA1 process ^4^I_11/2_ → ^4^F_7/2_ populated the ^4^I_11/2_ is not efficient. Therefore, the population to higher state is strongly governed by the ESA2 process, which populated ^4^F_9/2_ levels and yielding relatively intense red emission. As mentioned before, ETU3 is the main population process for green UC emission in LOS. Herein, all ETU process is less efficient due to the rapid relaxation of ^4^I_9/2_ and ^4^I_11/2_, leading to the G/R <1. Photons in LOSO tend to accumulate in the long-lived ^4^I_13/2_ state with an intense NIR emission at 1530 nm.

In conclusion, monodispersed LOS and LOSO nanocrystals with efficient MIR, NIR and visible UC emission have been successfully synthesized through a facile hydrothermal method with a subsequent gas-aided annealing process for the first time. The surfactant PVP plays an important role in control the morphology and size of the as-prepared precursor. Taking the advantage of the structural similarity, the as-prepared precursors convert to LOSO and LOS in air and CS_2_/CO atmosphere, respectively. Morphologies of the annealing LOS and LOSO nanocrystals with similar lattice structure and crystallinity maintain good spherical shape with an average diameter of ~300 nm at temperature as high as 900 °C, presenting an excellent thermal stability compared with other nanocrystal counterparts. Effect of annealing on morphology and fluorescent performance in MIR, NIR and visible UC has also been investigated in detailed. Due to the multiphonon relaxation, the visible UC and MIR emission intensity of LOS is much higher than that of in LOSO, while in NIR emission the situation is opposite. A possible energy transfer mechanism has been established to investigate the critical link between the multiphonon relaxation and the interionic interaction of Er^3+^ dopants. It is expected that the uniform LOS and LOSO nanocrystals with efficient fluorescent emission have potential applications in various fields including communication, chemical detecting, medical surgery and so forth.

## Method

### Precursor preparation

The synthesis of 5% Er^3+^-doped LOS and LOSO precursors was as follows. Rare earth oxide La_2_O_3_ and Er_2_O_3_ were purchased from Aladdin Reagent Co., Ltd., and were dissolved into the corresponding nitrite stock solutions by nitric acid in elevated temperature, respectively. Other chemicals were purchased from Sinopharm Chemical Regent Co., Ltd. All the starting chemicals are analytical grade and used directly without further purification. For the synthesis of precursors, 2.0 g poly(vinylpyrrolidone) (PVP K30, M = 40,000) were dissolved thoroughly into 25 mL of ethylene glycol (EG) under vigorous stirring. Then 3.9 mL of La(NO_3_)_3_ (0.5 M) and 0.25 mL of Er(NO_3_)_3_ (0.2 M) were added dropwise into the above solution. After keeping stirring for another 15 min, 10 mL of ethanol with excess thiourea (CN_2_H_4_S, 3 mmol) was added dropwise into the above mixture and stirred for another 1 h. The clear solution was then transferred into a 50 mL Teflon-line stainless autoclave and heated at 200 °C for 6 h. After naturally cooling to room temperature, the precursors were collected by centrifugation, washed with water and ethanol twice, respectively. Finally, the precursors were dried in atmosphere at 80 °C overnight.

### Heat treatment

LOSO and LOS nanocrystals were synthesized by calcination of the precursor in the atmosphere of air and CS_2_/CO, respectively. LOSO were calcined at 700–900 °C for 2 h in air with a heating rate of 3 °C/min. In order to reduce oxygen in atmosphere, LOS were calcined with a facile gas-aided sulfidation treatment, using CS_2_ and CO mixed gas as the sulfurization agents to replace H_2_S or N_2_/S which are toxic and normally easy to pollute the atmosphere furnace. In a typical procedure, 16 g sulfur (S) powder was mixed with excess activated carbon (C) in an corundum crucible sealed by borax (Na_2_B_4_O_7_·10 H_2_O) and pre-calcined at 800 °C to produce CS_2_/CO, which will be absorbed in vacancies within the activated C powder. Later on, the precursors were sealed in the above pre-calcined mixture and then heat at 700~900 °C for 2 h with a heating rate of 3 °C/min to obtain LOS nanocrystals.

### Characterization

The thermal analysis including thermogravimetry (TG) and differential scanning calorimetry (DSC) for the hydrothermal precursor powder was performed in a simultaneous thermal analyzer (STA449C, NETZSCH, Germany) under air flow with a heating rate of 10 °C/min. The X-ray diffraction (XRD) patterns were collected on an X’Pert PRO X-ray diffractometer (PANalytical, Netherland) with Cu Kα radiation (λ = 0.15418 nm). The morphologies of as-prepared precursors and resulted LOS/LOSO were performed by a Nova NanoSEM403 field emission-scanning electron microscopy (FE-SEM, FEI, Netherlands) with an accelerating voltage of 10 kV. Transmission electron microscopic (TEM) images, high-resolution TEM (HRTEM), selected area electron diffraction (SAED), high-angle annular dark-field scanning transmission electron microscopy (HAADF STEM) images and elemental mappings were investigated using HRTEM 2100 F (JEOL, Japan) equipped with energy-dispersive X-ray spectrometer (EDS). The MIR emission spectra were recorded on an OMNI5015i spectrometer (Zolix, China) with the excitation of a 980 nm laser diode (LD, LEO photoelectric, China). The corresponding MIR fluorescence decay times were also performed on the OMNI5015i spectrometer (Zolix, China) equipped with a TDS 3012B digital oscilloscope (Tektromix, USA) and a 980 nm pulsed LD (LEO photoelectric, China). The visible UC and NIR fluorescent spectra were obtained with an iHR 320 spectrometer (Jobin-Yvon, France) equipped with a 980 nm LD (LEO photoelectric, China) as excitation source. The iHR 320 spectrometer combined with the TDS 3012B digital oscilloscope (Tektromix, USA) and pulsed 808/980 nm LDs (LEO photoelectric, China) were also employed to measure the corresponding visible UC and NIR luminescence lifetimes of Er^3+^: LOS/LOSO. Raman spectra were collected by a Raman spectrometer (Renishaw in Via, UK) upon excitation of 532 nm laser. All the measurements were performed at room temperature.

## Additional Information

**How to cite this article**: Pan, Q. *et al*. Regulating Mid-infrared to Visible Fluorescence in Monodispersed Er^3+^-doped La_2_O_2_S (La_2_O_2_SO_4_) Nanocrystals by Phase Modulation. *Sci. Rep.* **6**, 37141; doi: 10.1038/srep37141 (2016).

**Publisher’s note:** Springer Nature remains neutral with regard to jurisdictional claims in published maps and institutional affiliations.

## Supplementary Material

Supplementary Information
