# Supplementary material for: Regulating Mid-infrared to Visible Fluorescence in Monodispersed Er3+-doped La2O2S (La2O2SO4) Nanocrystals by Phase Modulation
Source: Sci Rep. 2016 Nov 15;6:37141. doi: 10.1038/srep37141 (PMC5109033; doi:10.1038/srep37141)
Supplement: Supplementary Information [file srep37141-s1.pdf]

Supporting information

Regulating Mid-infrared to Visible Fluorescence in Monodispersed Er^{3+}-doped La_2O_2S ($La_2O_2SO_4$) Nanocrystals by Phase Modulation

Qiwen Pan[1], Dandan Yang[1], Shiliang Kang[1], Jianrong Qiu[2], Guoping Dong[1, *]

[1]State Key Laboratory of Luminescent Materials and Devices and Institute of Optical Communication Materials, School of Materials Science and Engineering, South China University of Technology, Guangzhou 510640, China

[2]State Key Laboratory of Modern Optical Instrumentation, College of Optical Science and Engineering, Zhejiang University, Hangzhou 310027, China

◆ **Corresponding authors:**

Prof. **Guoping Dong**

E-mail: dgp@scut.edu.cn

Tel.: +86-20-87114235

Address: State Key Laboratory of Luminescent Materials and Devices, South China University of Technology, Wushan Road 381, Guangzhou 510640, China.

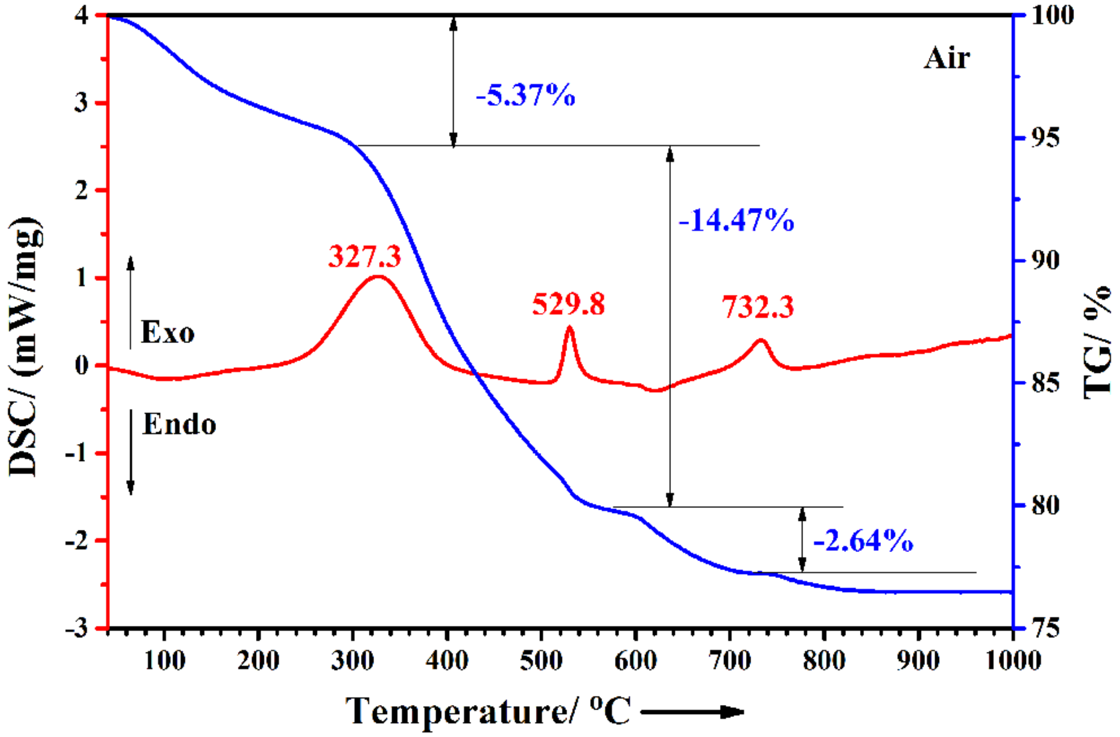

Fig. S1 Thermoanalytical (TG-DSC) curves of as-prepared 5% Er^{3+}-doped precursor under air flow with a heating rate of 10 °C/min.

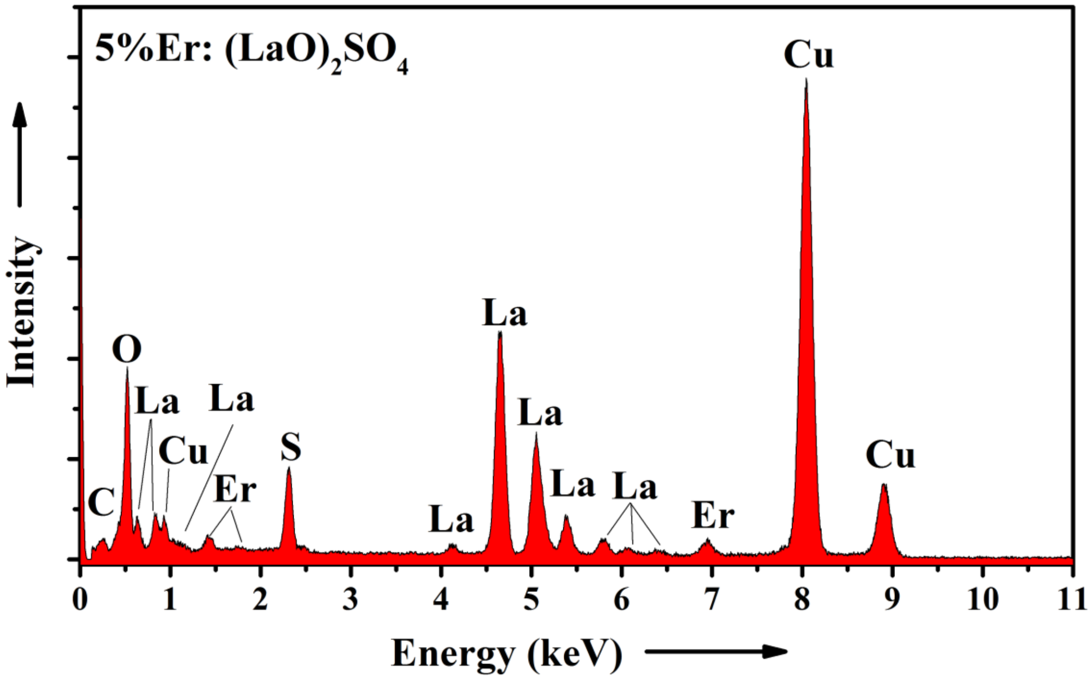

Fig. S2 EDS spectrum of 5% Er^{3+}-doped $La_2O_2SO_4$ nanocrystals calcined at 700°C. Elements of La, O, S and Er are observed in the spectrum. The peak of C is a part from the organic molecular in precursor, and a part from the copper grid. The signal of Cu only comes from the copper grid.

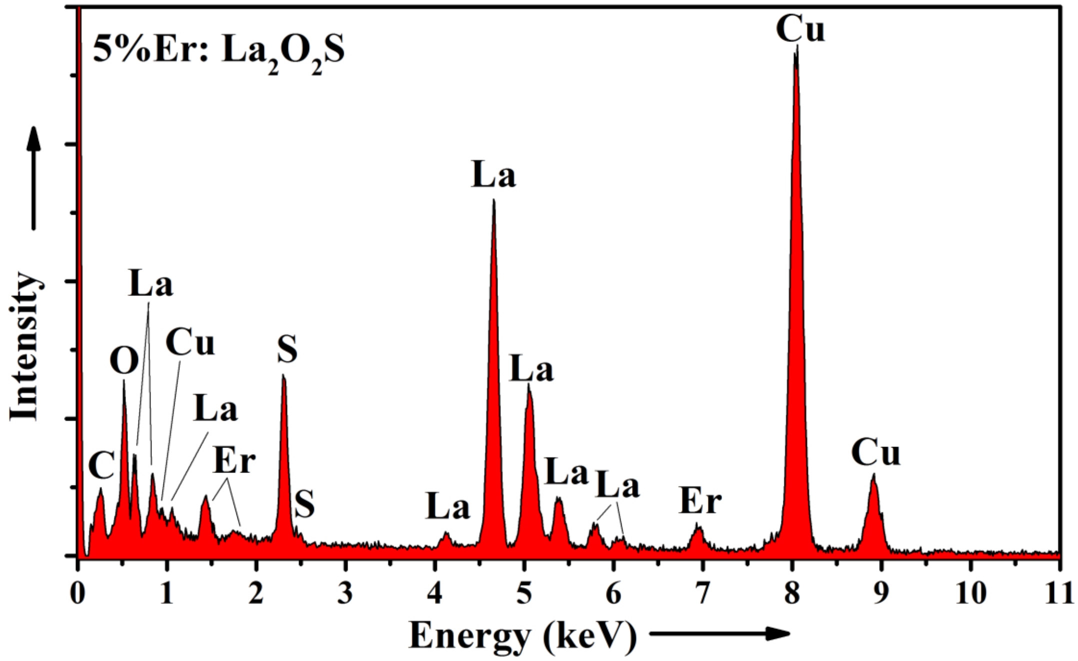

Fig. S3 EDS spectrum of 5% Er^{3+}-doped La_2O_2S nanocrystals calcined at 700°C. Elements of La, O, S and Er are observed in the spectrum. The peak of C is a part from the organic molecular in precursor, and a part from the copper grid. The signal of Cu only comes from the copper grid.
